# Authors’ Response to Peer Reviews of “The Association of Shared Care Networks With 30-Day Heart Failure Excessive Hospital Readmissions: Longitudinal Observational Study”

**DOI:** 10.2196/37005

**Published:** 2022-04-06

**Authors:** Diego Pinheiro, Ryan Hartman, Jing Mai, Erick Romero, Mohammad Soroya, Carmelo Bastos-Filho, Ricardo de Carvalho Lima, Michael Gibson, Imo Ebong, Julie Bidwell, Miriam Nuno, Martin Cadeiras

**Affiliations:** 1 Unicap-Icam International School Universidade Católica de Pernambuco Recife Brazil; 2 see Acknowledgments; 3 Department of Internal Medicine, Division of Cardiovascular Medicine University of California, Davis Sacramento, CA United States; 4 Polytechnic School of Pernambuco University of Pernambuco Recife Brazil; 5 Division of Cardiovascular Surgery University of Pernambuco Recife Brazil; 6 Family Caregiving Institute Betty Irene Moore School of Nursing University of California, Davis Sacramento, CA United States; 7 Department of Public Health Sciences, Division of Biostatistics University of California, Davis Sacramento, CA United States

**Keywords:** patient readmission, quality assurance, health care, catchment area, health, community networks, regional medical programs

*This is the authors’ response to peer-review reports for “The Association of Shared Care Networks With 30-Day Heart Failure Excessive Hospital Readmissions: Longitudinal Observational Study.”*

## Round 1 Review

### Reviewer BF [1]

#### General Comments

Thank you for the opportunity to review this study [2] of the association of shared care networks with heart failure (HF) excessive hospital readmissions. Hospital readmission is a very current topic. Nonetheless, several issues should be noted.

##### Authors’ Comment

We appreciate the recognition that HF excessive hospital readmissions is a very current topic.

#### Specific Comments

##### Major Comments: Comment 1

1. In “study population and design” in “methods,” the authors mentioned, “hospitals with less than 2 repeated measures of higher-than-expected HF readmission in the HRRP (Hospital Reduction Readmission Program) or without discharge data in the OSHPD (Office of Statewide Health Planning and Development) were excluded.” Does this mean this study only considered hospitals with repeated higher-than-expected HF readmission? Ignoring hospitals without repeated higher-than-expected HF readmission may introduce bias to the analysis. Please clarify why you have chosen this data inclusion criterion.

###### Authors’ Comment

We appreciate the comment about the exclusion of hospitals with less than 2 repeated measures and the bias such exclusion may produce. Given that the study design is longitudinal using generalized estimating equations (GEEs), repeated measures are required. Nevertheless, we rewrote this whole section, which is now as follows:

“Study Design, Study Setting, and Participants

This is an observational longitudinal study. The study setting was hospitals in California, US during the period from 2012 to 2017. Participants were all hospitals reported in the Hospital Readmissions Reduction Program (HRRP) (6). The eligibility criteria were as follows: At least 2 repeated measures of higher-than-expected HF readmission in the HRRP and availability of discharge data from the Office of Statewide Health Planning and Development (OSHPD) (16). These criteria enabled, respectively, carrying out a longitudinal study which requires repeated measures and linking data from the HRRP with date from OSHPD. Between 233 and 237 hospitals in California were included depending on the year. Ethical approval was unnecessary because all data was at the hospital-level and was already made publicly available from both HRRP and OSHPD. All code, processed data, built networks, and data analysis resulting from this work are available on the Open Science Framework (OSF) repository of this work (37).”

##### Major Comments: Comment 2

2. In “data sources” in “methods,” the authors collected excessive readmission ratio (ERR) data from 2012 to 2017. In almost every year, the HRRP updated the inclusion criteria of HF readmission (eg, lists of eligible diagnosis codes and procedure codes in the planned readmission algorithm). In this case, how did you fairly compare the ERR across different years?

###### Authors’ Comment

This is a very insightful comment and indeed requires extra discussion. The ERR is a risk-standardized 30-day readmission ratio. It is used by the HRRP to assess excess hospital readmissions and calculate hospital penalties [3]. The ERR has been used in longitudinal studies including the years of this study before [3-5].

The ERR is calculated by dividing the “predicted readmissions” (p) to “expected readmissions” (e). Using a hierarchical generalized linear model (HGLM), both “predicted” (p) and “expected” (e) readmissions are estimated using an “adjusted average intercept over all hospitals” (u), but the number of “predicted readmissions” (p), in addition, is estimated using a hospital-specific intercept deviation (a = u + w) from the “adjusted average intercept over all hospitals” (u). Such methodology, well documented in the Condition-Specific Readmission Measures Updates and Specifications Report from the Centers for Medicare & Medicaid Services (CMS) [6], makes the ERR an appropriate instrument for comparing hospitals within and between years.

The following text was included in “data sources” in “methods”:

“The ERR is calculated dividing the predicted readmissions to expected readmissions. Using a hierarchical generalized linear model (HGLM), both predicted and expected readmissions are estimated using an adjusted average intercept over all hospitals, but predicted readmissions, in addition, is estimated using a hospital-specific intercept deviation from the adjusted average intercept over all hospitals. Such methodology, well documented in the Condition-Specific Readmission Measures Updates and Specifications Report from the Centers for Medicare & Medicaid Services (CMS) [7], makes the ERR an appropriate instrument for comparing hospitals within and between years.”

##### Major Comments: Comment 3

3. Is the “Uncovering Shared Care Areas and Localization Index from Hospital-Patient Discharge Data” in “methods” a literature review of other studies or the method the authors used in this study? Please clarify. If it is a literature review, it should go in the “introduction.”

###### Authors’ Comment

Thank you for mentioning the methods in this subsection. Though it may appear to be a literature review, we are only specifying the parameters that were considered for each algorithm.

### Reviewer BX [7]

#### Major Comments: Comment 1

- Title: For this study, please include the type of study in the title. If you are considering 30-day readmission, please specify it in the title.

##### Authors’ Comment

We appreciate this comment, and following your suggestion, we changed the title to “Association of Shared Care Networks with 30-Day Heart Failure Excessive Hospital Readmissions: Longitudinal Observational Study.” We hope this new title is now appropriate.

#### Major Comments: Comment 2

- Abstract: Please move the objective section to the end of the background section, and it is recommended that it is written the same as in the study title.

##### Authors’ Comment

Thank you very much. Following your suggestion, we changed the objective to “This study aimed to evaluate the association of shared care networks with 30-day heart failure excessive readmission rates using a longitudinal observational study” to be written the same as the study title. We would love to move it to the end of the background section, but it seems that the Objective section is mandatory.

#### Major Comments: Comment 3

- Methods: Please start this section with the study design. Study setting, study variables, and outcomes and their measurements should be mentioned, briefly. Eligibility criteria have not been provided.

##### Authors’ Comment

Thank you for your suggestion. We rewrote the Methods section. Its first section is now “Study Design, Study Setting, and Participants.”

#### Major Comments: Comment 4

- Methods: ERR: I think it is excessive readmission risk ratio because no person-year has been reported. Thus, to improve the reporting, please revise it in the whole document.

##### Authors’ Comment

Thanks for the suggestion. We would rather use the same name used in the literature [6].

#### Major Comments: Comment 5

- Results: To facilitate the interpretation of the study results, please convert beta coefficients by exponentiating them.

##### Authors’ Comment

We understand the need of converting beta coefficients when dependent variables are dichotomous (binary). In our case, the ERR is not dichotomous but a continuous variable that can be less than or greater than 1 such as 0.92 or 1.23 depending on the presence or absence of excessive hospital readmissions. Therefore, we used a GEE with a Gaussian family without a Logit link function. In this case, we understand that converting the beta coefficients would not be appropriate because in their current form they express, on average, a 1-unit of change in the predictor variable.

We modified the text to clarify potential misunderstandings.

We included the following text in “data sources” in “methods”:

“The ERR is calculated dividing the predicted readmissions to expected readmissions. Using a hierarchical generalized linear model (HGLM), both predicted and expected readmissions are estimated using an adjusted average intercept over all hospitals, but predicted readmissions, in addition, is estimated using a hospital-specific intercept deviation from the adjusted average intercept over all hospitals. Such methodology, well documented in the Condition-Specific Readmission Measures Updates and Specifications Report from the Centers for Medicare & Medicaid Services (CMS) [6], makes the ERR an appropriate instrument for comparing hospitals within and between years.”

#### Major Comments: Comment 6

- Please use expanded forms of the abbreviations the first time they are mentioned. The expanded form of some abbreviations has not been provided.

##### Authors’ Comment

We appreciate this comment from the reviewer. The paper was revised to use the expanded form of the abbreviations for the first time. Additionally, we included all abbreviations in the Abbreviations section in alphabetic order.

“Abbreviations

ACS: American Community Survey

CMS: Centers for Medicare & Medicaid Services

ED: emergency department

ERR: excessive readmission ratios

HF: heart failure

HGLM: hierarchical generalized linear model

HRRP: Hospital Reduction Readmission Program

GEE: generalized estimating equations

LI: localization index

LVAD: Left Ventricular Assisted Devices

OLS: ordinary least squares

OSHPD: Office of Statewide Health Planning and Development

SCA: shared care area

STROBE: STrengthening the Reporting of OBservational studies in Epidemiology

OSF: Open Science Framework

UDS: Uniform Data System

ZCTA: ZIP Code Tabulation Area”

#### Major Comments: Comment 7

- Keywords: Please write these according to the Medical Subject Headings (MeSH) system.
- Introduction: The necessity of this study is not clear. Please provide a paragraph about the importance and necessity of this study and why you designed and conducted this study.

##### Authors’ Comment

We appreciate the encouragement to write keywords according to the MeSH system. We changed all our keywords as follows: “Patient Readmission; Quality Assurance, Health Care; Catchment Area, Health; Community Networks; Regional Medical Programs.”

#### Major Comments: Comment 8

- Methods: It is recommended to write this section according to the STROBE (Strengthening the Reporting of Observational Studies in Epidemiology) standard writing and refer to it in the first paragraph of the Methods section.

##### Authors’ Comment

Thank you for your suggestion. The first paragraph of the Methods sections now includes:

“This methods section was written according to the STrengthening the Reporting of OBservational studies in Epidemiology (STROBE) standard writing.”

Additionally, we changed the whole Methods section to include the following new subsections: Study Design, Study Setting, and Participants; Study Outcome; Study Variables; and Data Sources.

#### Major Comments: Comment 9

- Please start this section with the study design. A retrospective study is not a study design and refers to the type of data collection.

##### Authors’ Comment

Thank you for your suggestion. We rewrote the Methods section. Its first section is now “Study Design, Study Setting, and Participants.”

#### Major Comments: Comment 10

- Please provide information about institutional review board (IRB) approval of this study.

##### Authors’ Comment

Thank you for your concern. As we stated in the text, ethical approval was not necessary because all data used in this work is made publicly available by the HRRP and OSHPD.

#### Major Comments: Comment 11

- Study variables and their measurement should be provided.

##### Authors’ Comment

Thank you for your suggestion. The Methods section now has 3 new subsections: Study Design, Study Setting, and Participants; Study Outcome; and Study Variables, Data Sources.

#### Major Comments: Comment 12

- Statistical analysis: please use converted forms of beta coefficients.

##### Authors’ Comment

We understand the need of converting beta coefficients when dependent variables are dichotomous (binary). In our case, the ERR is not dichotomous but a continuous variable that can be less than or greater than 1 such as 0.92 or 1.23 depending on the presence or absence of excessive hospitals readmission. Therefore, we used a GEE with a Gaussian family without a Logit link function. In this case, we understand that converting the beta coefficients would not be appropriate because in their current form they express, on average, a 1-unit of change in the predictor variable.

We modified the text to clarify potential misunderstandings.

We included the following text in “data sources” in “methods”:

“The ERR is calculated dividing the predicted readmissions to expected readmissions. Using a hierarchical generalized linear model (HGLM), both predicted and expected readmissions are estimated using an adjusted average intercept over all hospitals, but predicted readmissions, in addition, is estimated using a hospital-specific intercept deviation from the adjusted average intercept over all hospitals. Such methodology, well documented in the Condition-Specific Readmission Measures Updates and Specifications Report from the Centers for Medicare & Medicaid Services (CMS) (17), makes the ERR an appropriate instrument for comparing hospitals within and between years.”

#### Major Comments: Comment 13

- Results: The Results section is very long. Please avoid providing data both in the text and the table.

##### Authors’ Comment

We understand the concern. The tables, however, contain more information than the text. In the text, we are providing some aspects of the results. We would prefer to keep the Results section without removing any text if possible.

#### Major Comments: Comment 14

- Please use converted forms of beta coefficients in the Results section.

##### Authors’ Comment

We understand the need of converting beta coefficients when dependent variables are dichotomous (binary). In our case, the ERR is not dichotomous but a continuous variable that can be less than or greater than 1 such as 0.92 or 1.23 depending on the presence or absence of excessive hospitals readmission. Therefore, we used a GEE with a Gaussian family without a Logit link function. In this case, we understand that converting the beta coefficients would not be appropriate because in their current form they express, on average, a 1-unit of change in the predictor variable.

We modified the text to clarify potential misunderstandings.

We included the following text in “data sources” in “methods”:

“The ERR is calculated dividing the predicted readmissions to expected readmissions. Using a hierarchical generalized linear model (HGLM), both predicted and expected readmissions are estimated using an adjusted average intercept over all hospitals, but predicted readmissions, in addition, is estimated using a hospital-specific intercept deviation from the adjusted average intercept over all hospitals. Such methodology, well documented in the Condition-Specific Readmission Measures Updates and Specifications Report from the Centers for Medicare & Medicaid Services (CMS) (17), makes the ERR an appropriate instrument for comparing hospitals within and between years.”

#### Major Comments: Comment 15

- Please identify adjusted and unadjusted beta coefficients in the Results section both in the Abstract and full text.

##### Authors’ Comment

Thank you for your review. We reviewed the manuscript and identified the adjusted and unadjusted beta coefficients.

#### Major Comments: Comment 16

- I do not think there is a “perspective section” in the JMIR structure. You can add it to the Discussion and Conclusion section if it is necessary.

##### Authors’ Comment

We apologize for including a perspective section. We moved it to the conclusion.

#### Major Comments: Comment 17

- Tables: They are not in the scientific form. Please revise them according to JMIR guidelines.

##### Authors’ Comment

Thank you for your comment. We apologize for not following the appropriate table style according to JMIR manuscripts. All tables were revised and should comply with JMIR standards.

## Round 2 Review

### Reviewer BX

I would like to thank the authors for considering all the reviewers’ comments.

However, there is no IRB or research ethics committee approval.

According to the authors’ statement “all data used in this work is made publicly available by the Hospital Reduction Readmission Program (HRRP) and Office of Statewide Health Planning and Development (OSHPD).” It is recommended to mention it in the Acknowledgments section and the first paragraph of the study design.

#### Authors’ Comment

We would like to thank the reviewer for all feedback provided. We agree with the reviewer. The current version of the manuscript now includes this sentence both in the Acknowledgments section and in the first paragraph of the study design.

## Abbreviations

CMS: Centers for Medicare & Medicaid Services
ERR: excessive readmission ratio
GEE: generalized estimating equation
HF: heart failure
HGLM: hierarchical generalized linear model
HRRP: Hospital Reduction Readmission Program
IRB: institutional review board
MeSH: Medical Subject Headings
OSF: Open Science Framework
OSHPD: Office of Statewide Health Planning and Development
REC: research ethics committee
STROBE: Strengthening the Reporting of Observational Studies in Epidemiology
